# Oculomotor nerve palsy recovery following microsurgery vs. endovascular treatment of posterior communicating artery aneurysms: a comparative meta-analysis of short- and long-term outcomes

**DOI:** 10.1007/s10143-024-03149-7

**Published:** 2024-12-18

**Authors:** Rahim Abo Kasem, Conor Cunningham, Sameh Samir Elawady, Mohammad Mahdi Sowlat, Sofia Babool, Saad Hulou, Zachary Hubbard, Atakan Orscelik, Basel Musmar, Alejandro M. Spiotta

**Affiliations:** 1 Department of Neurosurgery, Division of Neuroendovascular Surgery, Medical University of South Carolina, 96 Jonathan Lucas St, Charleston, SC 29425 USA; 2 Department of Neurology, Houston Methodist Hospital, Houston, TX USA; 3 University of Texas Southwestern Medical Center, Dallas, TX USA; 4 Department of Neurosurgery, University of Kentucky, Lexington, KY USA; 5 Department of Neurological Surgery, University of California, San Francisco, San Francisco, CA USA; 6 Department of Neurological Surgery, Thomas Jefferson University Hospital, Philadelphia, PA USA

**Keywords:** Oculomotor nerve palsy, Posterior communicating artery aneurysm, Microsurgical treatment, Endovascular treatment, Total recovery

## Abstract

Recent advancements in endovascular treatment (EVT) and different views on optimal management for posterior communicating artery (PComA) aneurysms with oculomotor nerve palsy (ONP) highlight a need to compare recovery timelines between microsurgery and EVT; heterogeneous outcomes and influencing factors may also affect results. A comprehensive systematic review and meta-analysis were conducted by searching PubMed, Embase, Scopus, and Web of Science databases. The extracted data encompassed patient demographics, details on treatment modalities and timing, and characteristics of PComA aneurysms ONP caused by either unruptured or ruptured aneurysms. The primary outcome was ONP favorable recovery, defined as the resolution of admission symptoms, except for subtle ptosis and mild pupillary asymmetry. We used random effect models to calculate odds ratios (OR) and pool prevalence with their corresponding 95% confidence intervals (CI). A total of 40 studies met the inclusion criteria. Overall, microsurgical clipping of PComA aneurysms demonstrated a significantly higher likelihood of ONP recovery compared to EVT at 1,3,6, and 12 months follow-up. However, recovery rates were comparable in long-term follow-up [18 months: (0.87 vs. 0.64, P-value = 0.36); ≥24 months: (0.86 vs. 0.72 P-Value = 0.26)]. The recovery outcomes for early treatment were similar when assessed during the 6-month follow-up (0.75 vs. 0.56, P-value = 0.07). Our findings suggest microsurgery leads to prompt ONP recovery from PComA aneurysms, while EVT shows potential for delayed favorable recovery; both treatments yield short-term recovery when administered early. A case-by-case approach is recommended, emphasizing a comprehensive understanding of patient factors in relation to the immediate and sustained effects of each treatment.

**Supplementary Information:**

The online version contains supplementary material available at 10.1007/s10143-024-03149-7.

## Introduction

Oculomotor nerve palsy (ONP) is a common presentation of both ruptured and unruptured posterior communicating artery (PComA) aneurysms [1–5]. Due to anatomical proximity, aneurysmal mass effect has been identified as the most prevalent ONP etiology and often leads to rupture [4, 6–8]. Therefore, microsurgery has been considered the preferred treatment due to prompt mass effect alleviation. However, recent literature has reported favorable ONP recovery outcomes after endovascular treatment (EVT) suggesting other mechanisms associated with ONP occurrence, such as the pulsatile hemodynamic stress (water hammer effect) of cerebral aneurysms [9, 10] as well as bleeding irritation in ruptured cases [5, 10, 11]. 

Complete ONP recovery depends on a multitude of factors such as the PComA aneurysm characteristics (rupture, size, location, direction, and type), the severity of ONP presentation, duration of symptoms, and treatment modality (Microsurgery, EVT, and emerging sub-techniques) [1, 3, 4, 7–9, 11–49]. Given these nuances, there is a lack of consensus on whether microsurgery or EVT provides more favorable ONP recovery outcomes in the setting of PComA aneurysms in the long term [25]. The timing from symptom onset to recovery may influence treatment outcomes, reflecting varied approaches and outcomes reported in the literature [11–49]. 

Thus, this updated meta-analysis aims to compare the time course of recovery outcomes of ONP induced by PComA aneurysms treated by microsurgery and EVT and the impact of early intervention on treatment outcomes, with a discussion of different factors at presentation.

## Methods

This systematic review was performed following the guidelines outlined in the Preferred Reporting Items for Systematic Reviews and Meta-Analyses (PRISMA) statements [50]. The Covidence software [51] was utilized to eliminate duplicate citations, screen titles and abstracts. Full texts were then evaluated.

### Search strategy

A literature search was conducted on PubMed, Web of Science, Embase, and Scopus until July 31, 2024, using the following keywords: (“Posterior Communicating Artery aneurysm*” OR “PComA aneurysm*” OR “PCOM aneurysm” OR “PCOMAA” OR “PCOMA”) AND ( “Oculomotor Nerve Palsy” OR “Oculomotor Nerve Paralysis” OR “Oculomotor Nerve Disease” OR “Oculomotor Nerve Disease Neuropathy” OR “Third Nerve Palsy” OR “3rd nerve Palsy” OR “third cranial nerve Palsy” OR “3rd cranial nerve Palsy” OR “ONP”). Additionally, a manual search was also conducted on the references of the included studies.

### Eligibility criteria and study selection

The literature screening was done by three reviewers (RAK, AO, and BM), and any discrepancies between the reviewers were resolved by discussion and consultation with the senior author (AS). We included studies that investigated patients of all ages who have ONP induced by PComA aneurysm and underwent treatment by either microsurgery or EVT. Studies were excluded if they did not report ONP recovery outcomes, or the definition put forth by authors was markedly unclear; in vitro studies, conference abstracts, expert opinions, case reports, anatomical or radiological studies, technical notes, or reviews were also excluded; in the case of overlapping studies, only the most recent and updated study was considered.

### Data extraction

Abstracts, full texts, and supplemental files of both comparative studies and single-arm studies were accurately searched by three independent reviewers (RAK, AO, and BM). Extracted data included: publication year, the country in which the study was conducted, study design, number of patients, demographics including age and sex, treatment (microsurgery or EVT), aneurysm-characteristics such as size, rupture, ONP-related variables including presentation (partial or total), time from symptoms onset until treatment, complications, time until favorable recovery, and recovery outcomes in each aforementioned subgroup and over various follow up intervals (1, 3, 6, 12, 18, ≥ 24-months).

### Study variables and outcome measures

Complete ONP diagnosis is established when the simultaneous occurrence of ptosis, fixed mydriasis, diplopia, and ophthalmoplegia is observed. Partial ONP is recognized by the absence of one or more of these symptoms. To address the need for unified criteria for ONP classification, the authors proposed a structured approach: categorization of patients into partial or total ONP, or complete recovery. When assessment measures were not explicitly detailed, the definition relied on terminologies used in the study, such as ‘recovery,’ ‘total/complete recovery,’ ‘no worsening,’ or ‘good recovery.’ Any discrepancies were resolved through consultation with a qualified ophthalmologist.

Complete ONP diagnosis is established when simultaneous occurrence of ptosis, fixed mydriasis, diplopia, and ophthalmoplegia are observed. Partial ONP is recognized by the absence of one or more of these symptoms. To address the need for unified criteria for ONP classification, the authors proposed a structured approach: categorization of patients into partial or total ONP, or complete recovery. When assessment measures were not explicitly detailed, the definition relied on terminologies used in the study such as “recovery,” “total/complete recovery,” “no worsening,” or “good recovery.” Any discrepancies were resolved through consultation with a qualified ophthalmologist.

The primary outcome was a favorable ONP recovery, defined by the resolution of all initial symptoms, except for subtle ptosis and mild pupillary asymmetry. We performed subgroup analyses for this outcome: (1) by aneurysm rupture status, (2) by age (< 60, ≥ 60) (3) by timing of treatment (early; within 7 days after the ONP onset, late; > week), (4) by aneurysm size (greater diameter): small aneurysms (≤ 7 mm) and large aneurysms (> 7 mm). The primary investigator and neurosurgeons supervising the study determined the cutoffs for age, treatment timing, and aneurysm size. These decisions were based on a detailed review of the included studies, identifying common criteria consistently reported across them and published literature. This process ensured that the thresholds were both clinically significant and aligned with established evidence.

### Risk of bias and quality assessment

Two reviewers independently assessed the risk of bias in the included studies using the Newcastle-Ottawa Scale (NOS) for cohort studies [52]. Studies that received a rating of 7 or more stars out of 9 were classified as high quality (low risk of bias), those with 5–6 points as moderate quality, and studies with 5 or fewer stars as low quality.

For the selection domain, studies received one point if the comparison cohorts were representative of the average patients receiving treatment for PComA aneurysms with ONP in the community, and another point if the cohorts were captured from the same population, such as patients treated at the same hospital or institution. Points were also awarded for the ascertainment of exposure and for demonstrating that the outcome of interest was not present at the start of the study.

In the comparability domain, studies received 2 points if they matched or statistically controlled for key factors, such as baseline characteristics, comorbidities, aneurysm size, and the number of ruptured aneurysms, ensuring that differences in outcomes could be attributed to the treatment modality rather than patient characteristics. For non-comparative studies, a modified version of the NOS was used [52, 53]. This modified NOS has a total of 8 points, with specific adjustments to the comparability domain. In the modified version, only one point was awarded for comparability, based on a comparison of subgroups, such as different aneurysm rupture statuses.

For the outcome assessment domain, studies received one point if the primary outcome, defined as favorable recovery from ONP, was adequately assessed, another point if the follow-up period was sufficient (at least 6 months), and one more point if there was minimal loss of subjects during follow-up, with adequate accounting in the analysis.

Disagreements between the two reviewers regarding the quality assessment were resolved through discussion and consensus.

### Statistical analysis and results synthesis

The meta-analysis was conducted using R software version 4.3.2 (RStudio Inc., MA, USA), employing the **meta** and **metafor** packages for statistical calculations, **effsize** for effect size metrics, and **forestplot** and **ggplot2** for visualizations. We pooled prevalence for single-arm studies and calculated odds ratio (OR) for double-arm studies with 95% confidence intervals (CI). Testing for heterogeneity was carried out using Q statistics, χ2, and I^2^ measures [54] with heterogeneity considered significant when I^2^ exceeded 50% or if the p-value was less than 0.05. Given the presence of methodological heterogeneity among the included studies, we employed a random effects model to amalgamate the data [55]. Funnel plots or the Egger test were utilized to assess the risk of publication bias in subgroups comprising more than 10 studies. We conducted a leave-one-out sensitivity analysis to evaluate the impact of individual studies on the overall results, and no substantial effect on the results was observed. The studies in our review were of similar design, and excluding low-quality studies did not significantly alter the outcomes, reflecting minimal variability in study quality.

## Results

### Search results

**Fig. 1:**
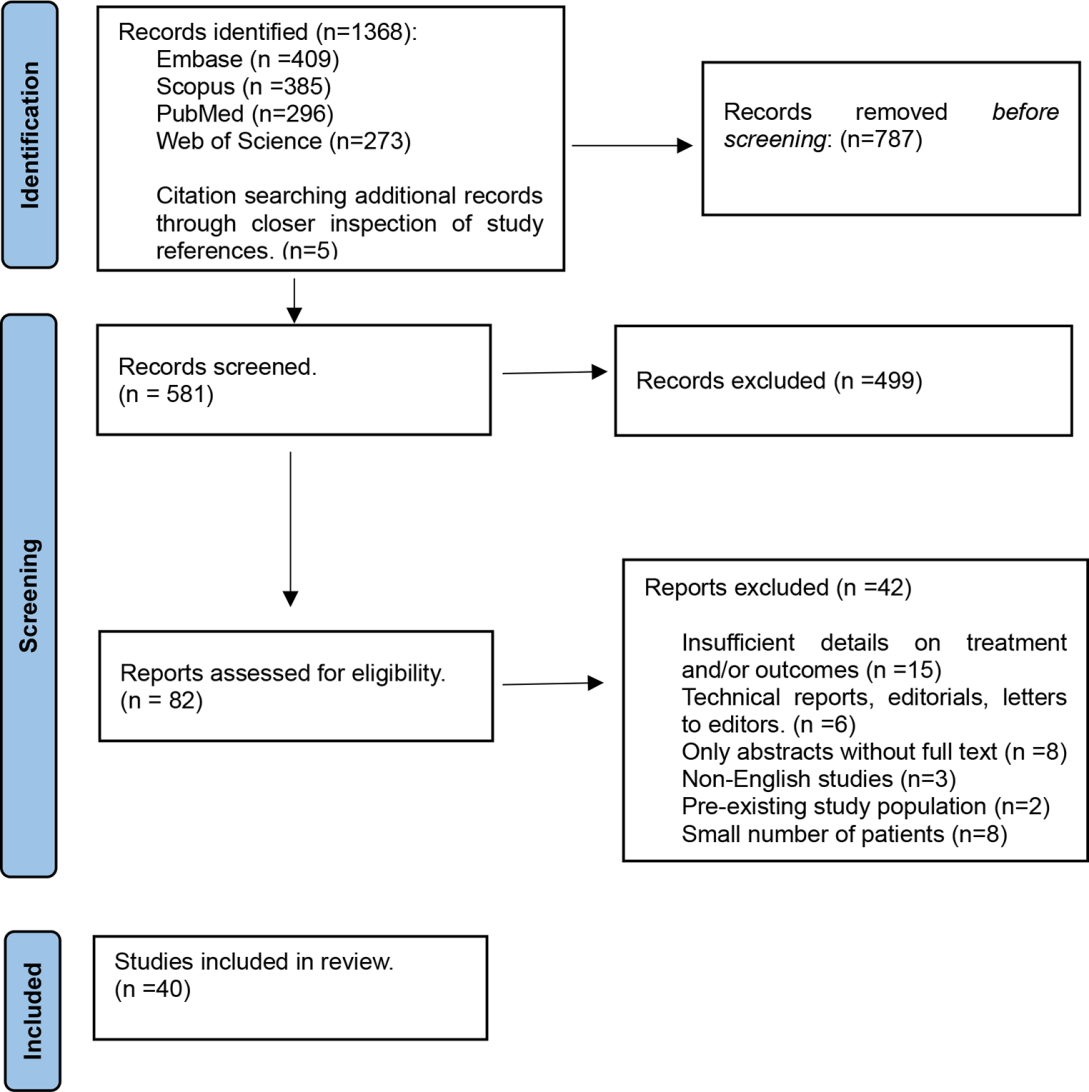
A flow diagram describing our comprehensive literature search

The comprehensive literature search yielded 581 studies. After screening the titles, and abstracts, and evaluating them in full text, 40 studies were included in our analysis (Fig. 1). Among these, 21 studies compared the outcomes of microsurgery and EVT, while 15 reported outcomes of EVT only, and 4 studies included patients who underwent microsurgery.

### Baseline characteristics of studies

A total of 1,964 patients were included, as detailed in Supplemental material 1, Table 1, Supplemental material 2, Table 2. All studies followed a retrospective cohort design. Within the study cohort, 1,040 (52.95%) patients underwent microsurgery, while 924 (47.05%) received EVT. The average age of patients ranged from 49 to 73 years in both treatment groups and 73% were females. The average aneurysm size varied between 6 and 10.5 mm.

### Quality assessment

All the included matched cohort studies had moderate to high quality. The quality assessment is summarized in Supplemental Material 1, Table 1, Supplemental Material 2, Table 2.

### Outcomes

#### Overall ONP recovery and treatment complications

**Fig. 2:**
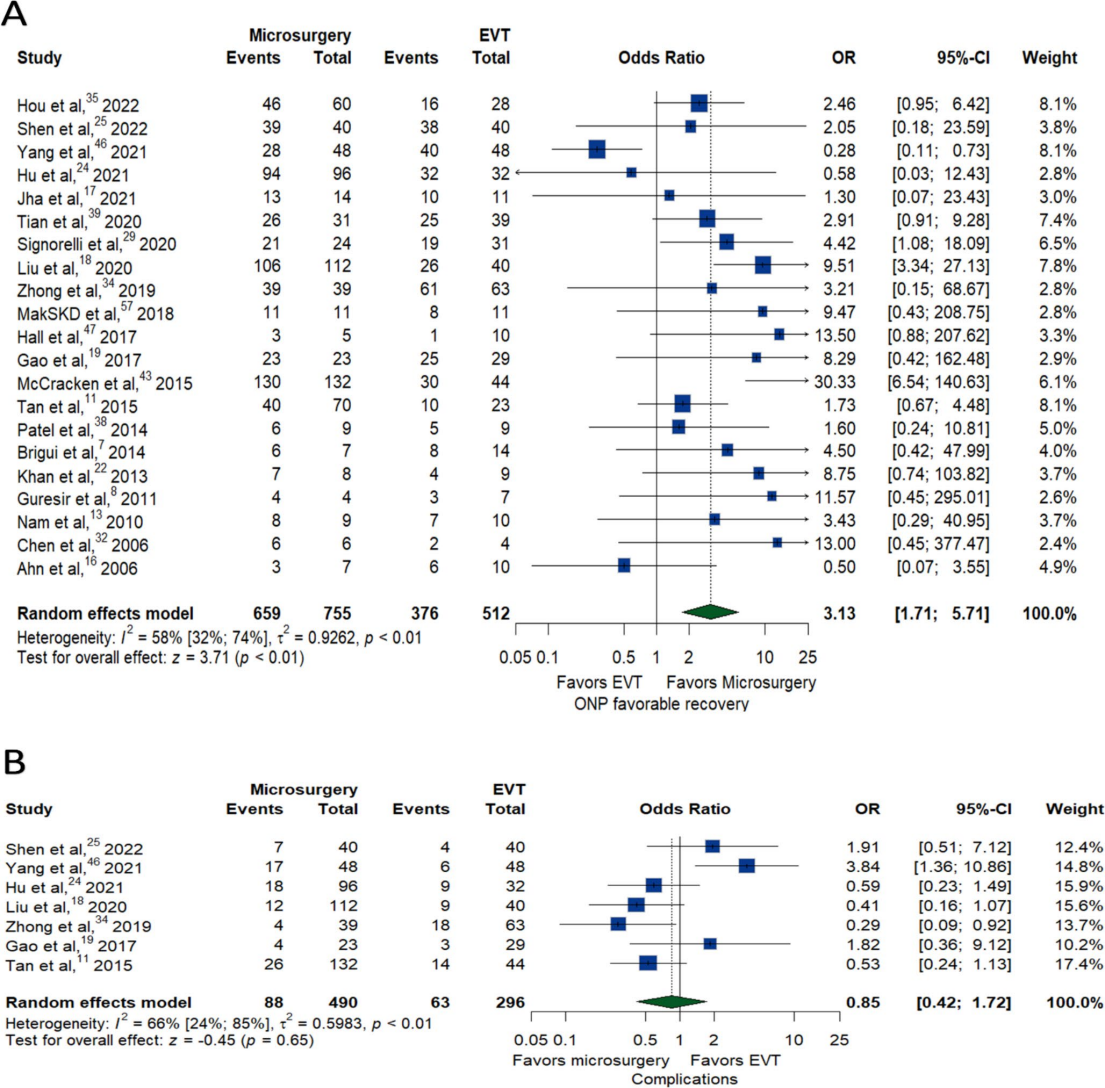
Forest plots of favorable ONP recovery outcomes (**A**) and complications (**B**) between microsurgery and EVT groups

Microsurgery demonstrated significantly higher favorable ONP recovery in the last indicated follow-up compared to EVT (OR: 3.13, 95% CI:1.71–5.71, *P* < 0.01) (Fig. 2A). A meta-analysis of seven studies with 490 patients who had microsurgery and 296 patients who had EVT found no significant difference in complication rates between the two groups. (17.96% for microsurgery, 21.28% for EVT, OR: 0.85, 95% CI: 0.42–1.72; *P* = 0.65). However, there was significant heterogeneity among these studies (I^2^ = 66%, *p* < 0.01), with more recent studies favoring EVT (OR:3.23, 95% CI:1.02–8.21, *P* < 0.01) (Fig. 2B).

#### Timecourse of ONP recovery

**Fig. 3:**
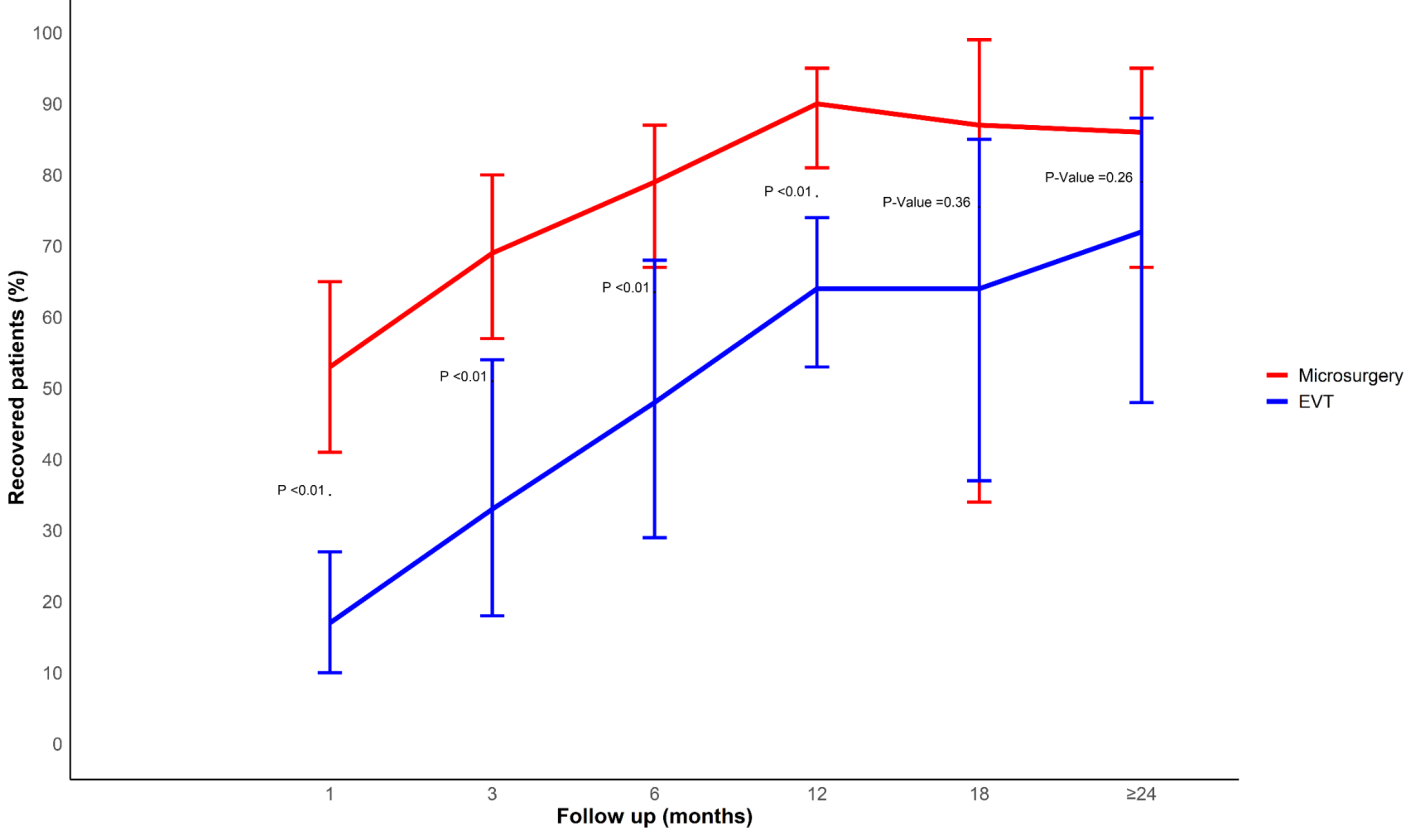
Timecourse comparison of the recovery of Oculomotor Nerve Palsy induced by PComA aneurysms for microsurgery versus EVT groups– Pooled data from comparative and non-comparative studies

The proportions of favorable ONP recovery were higher in microsurgery compared to EVT at 1 month (0.53 vs. 0.17, *P* < 0.01), 3 months (0.69 vs. 0.33, *P* < 0.01), 6 months (0.79 vs. 0.48, *P* < 0.01), and 12 months follow-up (0.90 vs. 0.64). However, beyond 12 months, recoveries between microsurgery and EVT became comparable (18 months: (0.87 vs. 0.64, P-value = 0.36; ≥24 months: 0.86 vs. 0.72 P-Value = 0.26) (Fig. 3).

#### Early versus delayed treatment

**Fig. 4:**
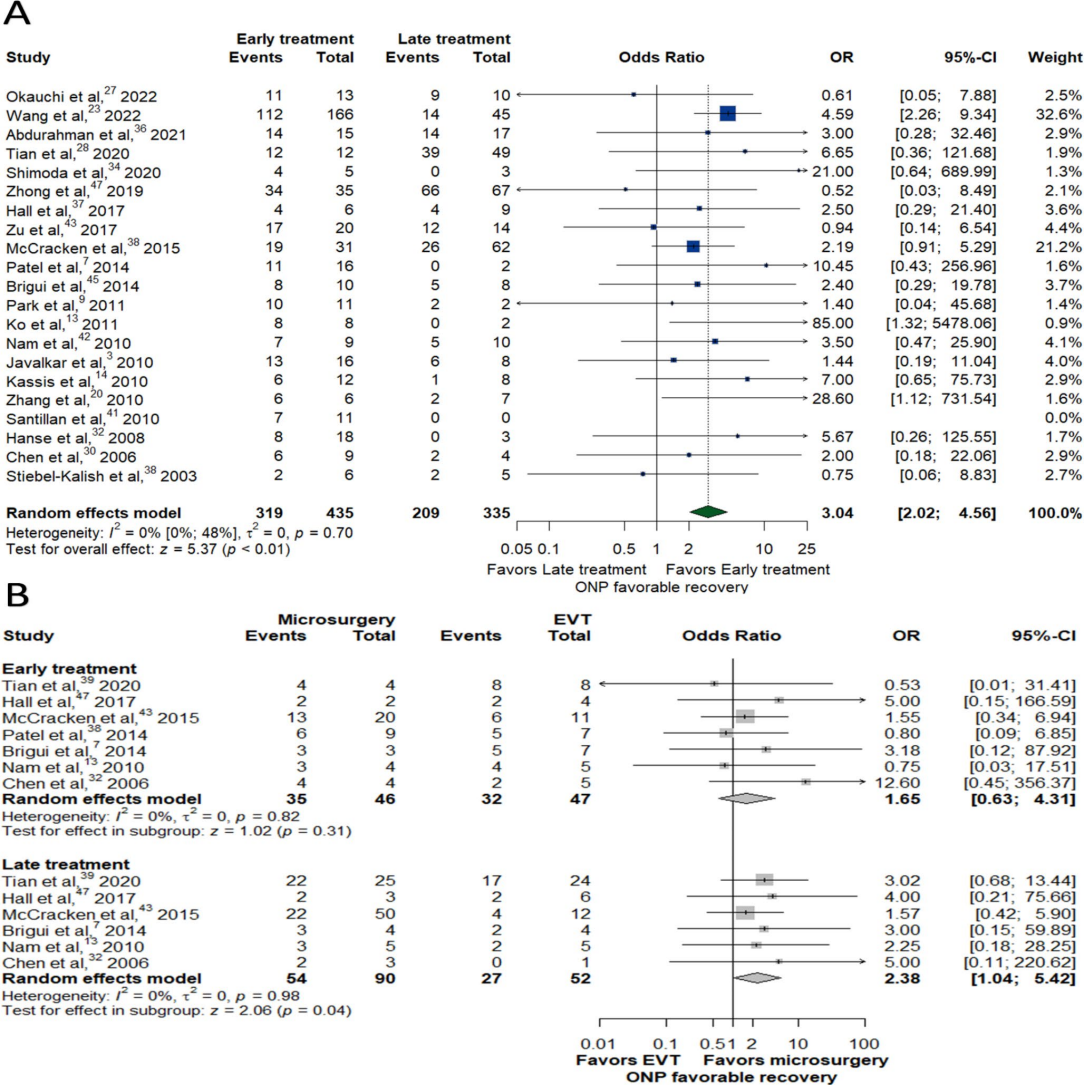
Forest plots of favorable recovery outcomes for the early time window group (≤ 7 days) compared with the late treatment window group (> 7 days) (**A**). And subgroup analysis of favorable ONP recovery outcomes comparing microsurgery with EVT in each treatment window group (**B**)

**Fig. 5:**
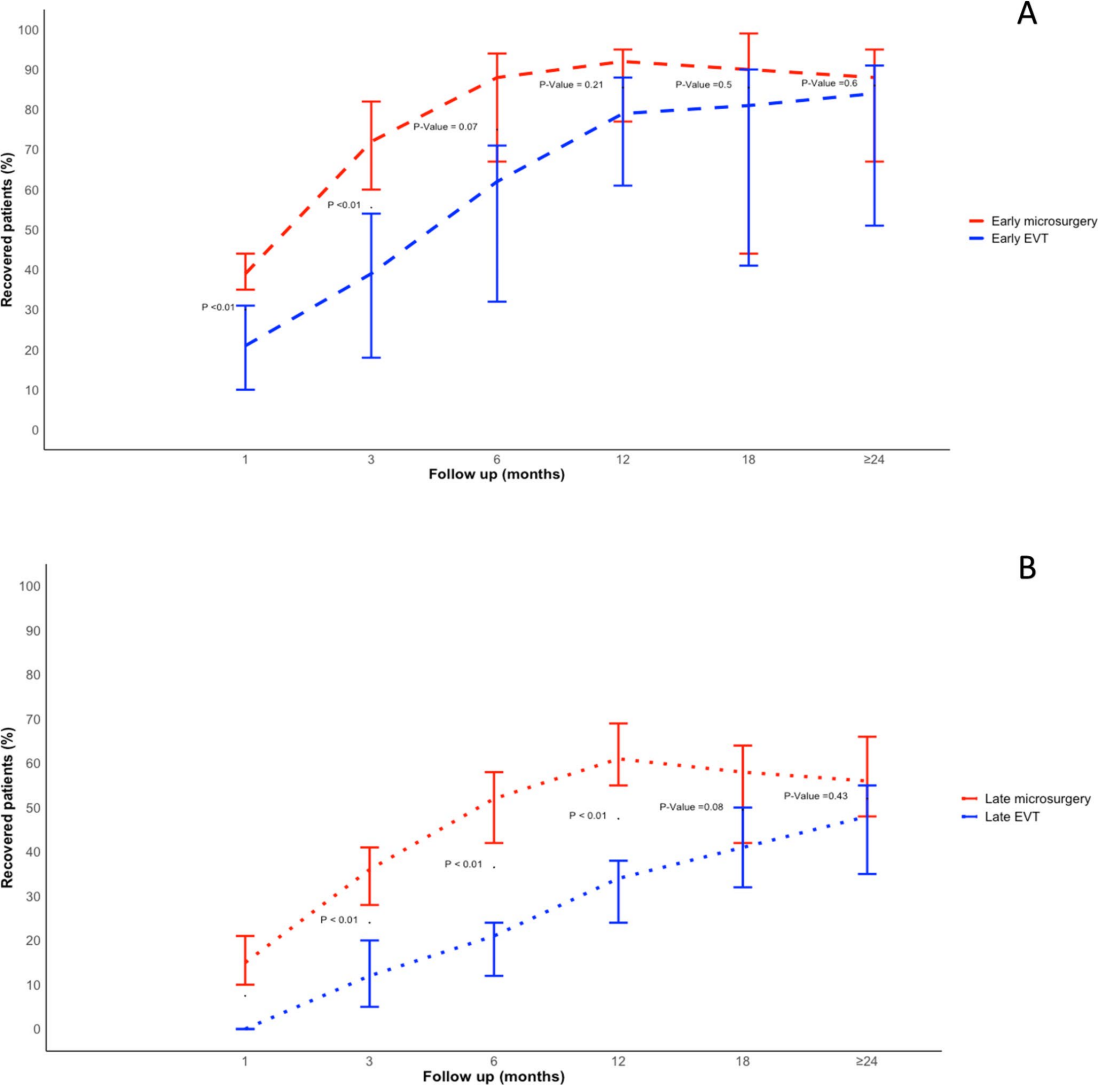
Timecourse comparison of the recovery of Oculomotor Nerve Palsy induced by PComA aneurysms for microsurgery versus EVT groups in early treatment window (≤ 7 days) (**A**) and late treatment window (> 7 days) (**B**)– Pooled data from comparative and non-comparative studies

Delayed treatment was associated with significantly lower rates of favorable recovery, regardless of the treatment (OR: 3.04, 95% CI:2.02–4.56, *P* < 0.01) (Fig. 4A). Among those treated in the early time window group (≤ 7 days from symptoms onset), there was no significant difference in recovery rates between microsurgery and EVT groups (OR = 1.65, 95% CI = 0.63–4.31, *P* = 0.31) (Fig. 4B). After analyzing the recovery timeline, the proportions of favorable ONP recovery were comparable in microsurgery compared to EVT starting at 6 months (0.75 vs. 0.56, P-value = 0.07) (Fig. 5A), However, microsurgery was associated with significantly higher favorable recovery rates in the late treatment window group (OR = 2.38, 95% CI = 1.04–5.42, *P* = 0.04) (Fig. 4B) with delayed comparable outcomes beyond 18 months follow up (0.59 vs. 0.41, P-value = 0.08) and ≥ 24 months follow up (0.56 vs. 0.45, P-value = 0.43) (Fig. 5B).

#### Factors associated with ONP recovery and association with treatment modality

**Fig. 6:**
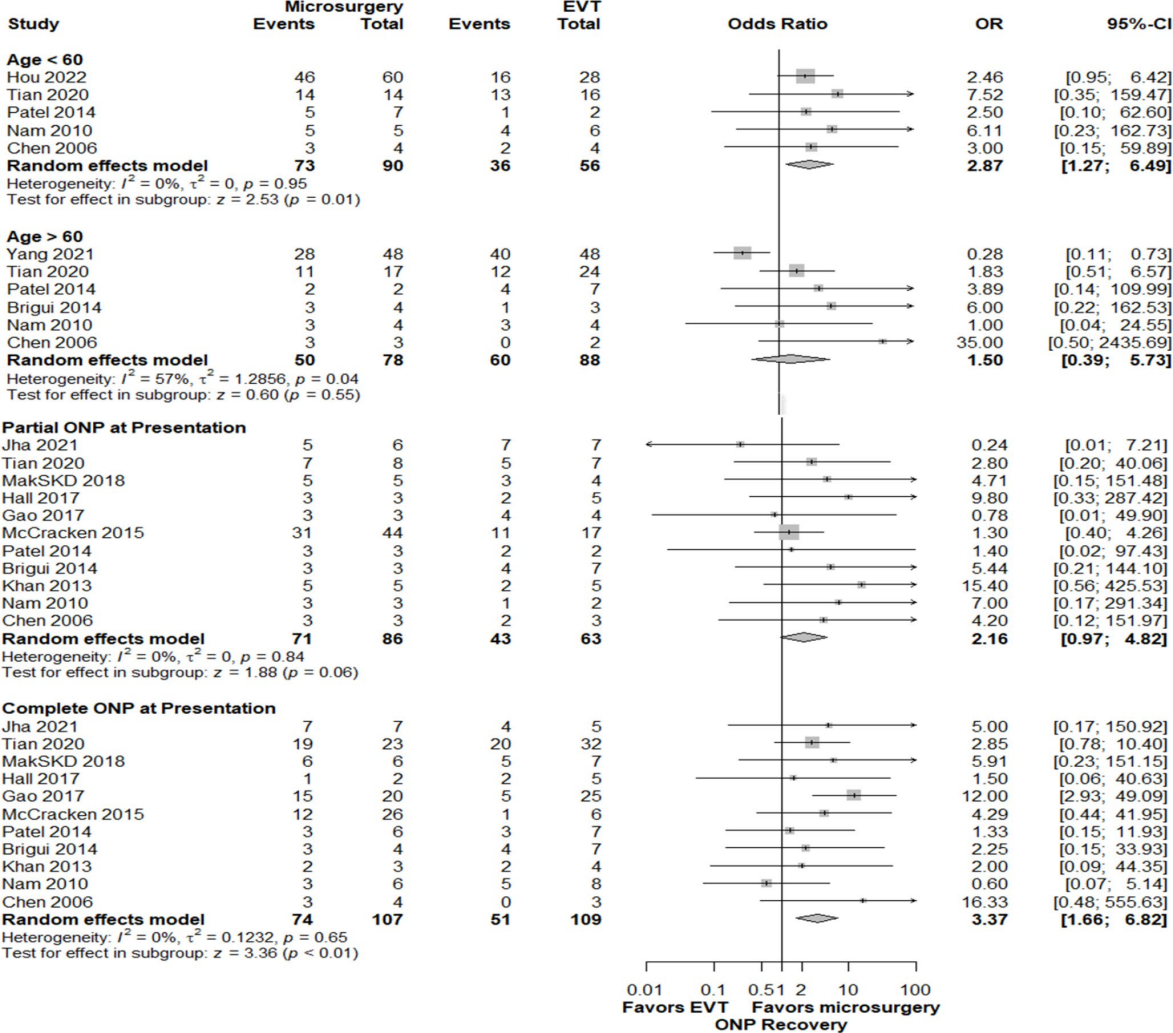
Favorable ONP recovery outcomes comparing surgical treatment with endovascular interventions in Age groups and ONP status at presentation groups

**Fig. 7:**
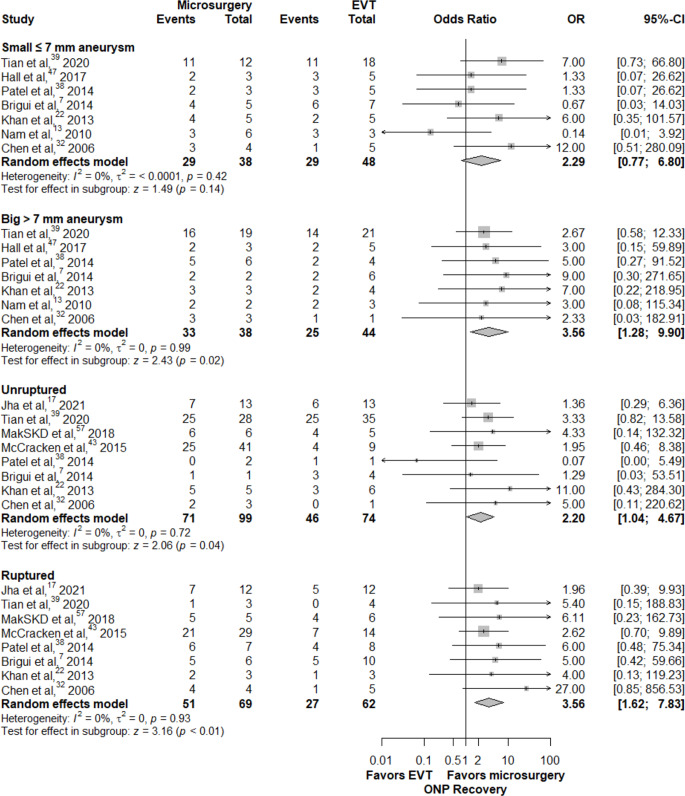
Favorable ONP recovery outcomes comparing surgical treatment with endovascular interventions in aneurysm size and rupture status groups

There was no significant difference in the ONP recovery between patients < 60 and ≥ 60 years old patients (71% vs. 69%, *P* = 0.76) presenting with ruptured aneurysms compared with those presenting with unruptured aneurysms (67.7% vs. 59.3%, *P* = 0.39), or between small (≤ 7 mm) and large (> 7 mm) aneurysms (65% vs. 64%, *P* = 0.88). Patients presenting with total ONP had significantly lower rates of good recovery outcomes than those with partial ONP (55% vs. 72%, *P* < 0.01). (Supplemental content 3, Table 3) Among patients < 60 years of age, those who underwent microsurgery exhibited a higher recovery rate (OR = 2.87, CI = 1.27–6.49, *P* = 0.01) while there was no significant difference between microsurgery and EVT in patients ≥ 60 years (OR = 1.50 CI = 0.39–5.73, *P* = 0.55). For the total ONP group, microsurgery showed significantly higher recovery rates when compared to EVT (OR: 3.37, 95% CI:1.66–6.82, *P* < 0.01). In the partial ONP group, there was no significant difference (OR: 2.16, 95% CI:0.97–4.82, *P* = 0.06) (Fig. 6). Both ruptured and unruptured aneurysm subgroups showed that microsurgery had significantly better recovery rates (OR: 3.56, 95% CI:1.62–7.83, *P* < 0.01 and OR: 2.20, 95% CI: 1.04–4.67, *P* = 0.04 respectively). In the subset of small aneurysms (≤ 7 mm), there was no significant difference in recovery rates between microsurgery and EVT groups (OR: 2.29, 95% CI:0.77–6.80, *P* = 0.14). For large aneurysms (> 7 mm), microsurgery showed significantly higher recovery rates (OR: 3.56, 95% CI:1.28–0.90, *P* = 0.02) (Fig. 7).

Meta-analysis of pooled data from both comparative and non-comparative studies demonstrated similar outcomes when comparing microsurgery versus EVT, except in cases of rupture (73.9% Vs 66.4% *P* = 0.23) (Supplemental material 3, Table 3).

## Discussion

In recent years, significant advancements in endovascular techniques have transformed the landscape of treatment options for patients with posterior communicating artery aneurysms [56, 57]. These improvements have led to increased interest in whether endovascular approaches could offer benefits comparable to or even better than traditional microsurgery; this was especially novel, considering the preference for less invasive procedures by patients and physicians. Our study compared microsurgery and EVT outcomes of PComA aneurysms presenting with ONP. Specifically, we assessed the time course of favorable recovery rates of ONP following treatment. According to the meta-analysis of follow-up results, microsurgery was associated with higher rates of favorable ONP recovery outcomes at 1, 3, 6, and 12-month follow-ups. Notably, after 12 months, comparable recovery outcomes were identified. In a subgroup analysis of early treatment window patients, comparable recovery was achieved earlier at 6 months follow-up. Microsurgery had higher rates of favorable ONP recovery compared to EVT in < 60-year-old patients, large aneurysms (≥ 7 mm), ≥ 7 days of ONP symptoms, and in cases of total ONP. Among ruptured aneurysms, partial ONP, small aneurysms, ≥ 60-year-old patients, and with early treatment, favorable ONP recovery outcomes of microsurgery and EVT were comparable.

### Timecourse of ONP recovery outcomes

Understanding the detailed timelines of oculomotor nerve palsy (ONP) recovery, both in the short and the long term, is essential for clinical decision-making and patient counseling, as the timing of recovery can significantly impact a patient’s quality of life and functional outcomes [44]. This study addresses this gap by offering an updated analysis that specifically examines the recovery trajectories following both microsurgical and endovascular interventions. Other meta-analysis studies, with less than 12 months follow-up duration, found lower overall recovery rates, and significantly higher ONP recovery rates associated with microsurgery compared to EVT [5, 43], which is consistent with the findings in our study. However, in this present meta-analysis, increasingly favorable ONP recovery was achieved with a longer duration of follow-up; this would presumably enable fibrosis and shrinkage of the treated aneurysm [6]. The meta-analysis revealed distinct recovery patterns for microsurgery and EVT over time. Microsurgery remains a well-established treatment modality for alleviating ONP symptoms, demonstrating high efficacy in achieving immediate symptom resolution. However, recent evidence indicates that EVT may also contribute to favorable outcomes in ONP management. Although the immediate postoperative recovery rates of EVT are comparatively modest, there is a notable, steady increase in recovery proportions observed over time. This suggests that EVT facilitates a gradual improvement in ONP symptoms, potentially leading to substantial recovery over time. Importantly, our study found that early intervention with EVT may accelerate this recovery process, highlighting its potential role as a viable alternative to traditional surgical methods, particularly in cases where microsurgery is not feasible or poses higher risks, or in cases of patients and physician preferences of less invasive intervention.

In the short term, microsurgery is traditionally preferred, as it effectively eliminates the compressive damage of the aneurysm to the oculomotor nerve; this alleviates the symptoms promptly, and stops the ongoing repetitive injury of arterial pulsation [5, 45, 46]. Of note, detailed surgical technique data is lacking as to whether or not the aneurysms were being needle punctured following clip application.

Although there are concerns with EVT, such as the contribution to mass effect with coiling, recent studies report remarkably high rates of complete nerve recovery (50–85%) with EVT [9, 14, 23, 27, 49]. EVT may not result in a quicker favorable recovery of ONP, but in long-term follow up, it will ultimately lead to a similar ONP recovery rate as microsurgery. This can be attributed to the elimination of pulsation and the long-term shrinking of the treated aneurysm leading to advantages beyond immediate relief [25]. 

These findings suggest that treatment decisions should be personalized, taking into account both short-term and long-term outcomes to weigh the risks and benefits for each individual.

### Early versus delayed window treatment

A crucial factor influencing ONP recovery time from is the duration of symptoms prior to treatment. Research indicates, consistent with our findings, that a shorter symptom duration—defined as 14 days or less—prior to intervention significantly enhances the likelihood of complete recovery [17, 23], highlighting the importance of prompt management. Signorelli et al. conducted a multicenter cohort study of patients with unruptured PComA aneurysms and found that the key predictor of ONP recovery was timely, complete, and durable aneurysm exclusion, irrespective of the treatment approach employed [29]. These findings align with our study, which demonstrates that patients receiving early treatment have a three-fold increased odds of achieving complete recovery compared to those treated later. Moreover, early intervention not only improves the likelihood of complete recovery, but also accelerates the time course of recovery. In this subgroup, ONP early recovery rates are comparable between surgical and endovascular interventions, surpassing the outcomes observed in the main meta-analysis and those treated during the delayed window. However, in both early and delayed treatment windows, microsurgery remains superior to endovascular therapy in terms of ONP recovery outcomes.

### Factors influencing treatment outcomes: considerations for optimal decision-making

It is crucial to note that these findings can be better interpreted if various major factors influencing treatment outcomes are considered, when viewed in the context of our meta-analysis and systematic reviews of the literature. As it seems to rapidly relieve the compressive effect [5–8, 47], microsurgery is the optimal treatment for patients with larger PComA aneurysms that may induce a stronger mass effect- the predominant cause of total ONP- [1, 5] as well as in cases of prolonged ONP symptoms that suggest severe nerve damage. Such factors related to nerve injury appear to play a stronger role in recovery [17]. A longer follow-up time is thus necessary for patients with a longer history of ONP or total ONP at presentation, as full recovery may take up to 2 years after microsurgery [3]. However, this has not been explored in EVT.

EVT of PComA aneurysms can also relieve ONP by eliminating the pulsation effect in the short term, yielding comparable recovery rates in cases with less severe mass effect; this may include cases such as partial palsy with reversible nerve damage, small PComA aneurysms, and shorter time from ONP onset to treatment [2, 6]. To address the inconsistency in partial ONP evaluation, future research could implement more efficient and accurate measures to better explore nerve injury extent and optimize outcomes for both treatments modalities. On the other hand, the correlation between aneurysm size and risk of developing ONP [47] has been questioned because this complication can occur also in the context of very small aneurysms [58]. Thus, our meta-analysis concluded that aneurysm size cannot be considered a predictive factor for ONP recovery. However, in small aneurysms ≤ 7 mm, EVT could yield comparable results to microsurgery.

In ruptured cases, the irritative effects of subarachnoid blood and its resultant inflammation may also contribute to nerve damage resulting in ONP, as opposed to unruptured cases where preexisting mass effect is the sole cause of ONP [3, 15, 37]. These patients tend to show early recovery from ONP due to the prompt attention they receive, leading to earlier treatment. Moreover, both microsurgery and EVT offer protection against rebleeding [4, 5, 7, 37, 45]. However, microsurgery removes surrounding blood immediately [5, 7]. 

EVT could specifically be preferable for patients ≥ 60 years old and ineligible for microsurgery by effectively reducing potential complications and promoting the recovery of nerve function [46, 59]. Few studies reported complication rates of each treatment modality [11, 18, 19, 24, 25, 34, 46]. Overall, the rate of complications is low, and neither confers a benefit over the other by complication rate alone.

While EVT and microsurgery have been generally studied, the comparison of their effects on ONP recovery within specific subgroups remains underexplored. This gap highlights the need for targeted research to better understand how these factors influence recovery outcomes across different treatment modalities.

### Limitations

With a large sample size, our meta-analysis represents a pioneering effort, underscoring the significance of incorporating timing and follow-up in the assessment of treatment efficacy and identifying key factors influencing recovery. Nonetheless, the present study has several limitations. First, the methodologic quality of the included studies along with the variables and outcome measures were not constant. Our results were also associated with the possibility of publication bias. Second, the advancement of surgical [5, 12, 45] and endovascular [4, 23, 27] techniques necessitates further investigation to optimize therapeutic strategies. Our analysis found that the broad classification of “EVT” lacked granularity to assess outcome differences between primary coiling, stent-assisted coiling, and flow diversion. Most studies reported coiling without specifying stent usage, and flow diversion was infrequently detailed, precluding a robust analysis of technique-specific outcomes. Third, because all the included studies are retrospective and non-comparative, meaningful direct comparisons are limited. Finally, data on important factors such as anatomical variations, morphological-hemodynamic patterns (wall shear stress [31], aneurysmal direction [23, 36], and the distance between the internal carotid artery and the anterior–posterior clinoid process [48]) and anti-inflammatory medications [23] were not available and thus, we were unable to control for them. Additionally, our study lacked a comparison of subgroups over time. We did not have sufficient data to analyze these timepoints accurately. Future prospective studies should validate the clinical impact of various surgical approaches, intrasaccular and extrasaccular endovascular techniques, and account for different impacting factors.

## Conclusion

Our findings indicate the superiority of microsurgery in prompt ONP recovery induced by PComA aneurysms and potentially comparable rates of delayed favorable ONP recovery outcomes associated with EVT. Within the early treatment window, comparable short-term ONP recovery can be achieved in both treatments. EVT also demonstrated comparable outcomes to microsurgery in cases of partial ONP, ruptured PComA aneurysms, age ≥ 60, and small aneurysms.

A case-by-case approach is recommended when selecting the optimal treatment modality, emphasizing a holistic understanding of all relevant factors for favorable recovery outcomes. This perspective contributes to the customization of treatment decisions based on warranted immediate and sustained effects.

## Electronic supplementary material

Below is the link to the electronic supplementary material.

Supplementary Material 1

## Data Availability

Template data collection forms; data extracted from included studies; data used for all analyses; analytic code; any other materials used in the review are available upon request from the corresponding author.

## Abbreviations

ONP: Oculomotor Nerve Palsy
PComA: Posterior Communicating Artery
EVT: Endovascular Therapy
PRISMA: Preferred Reporting Items for Systematic Reviews and Meta-Analyses ()
CI: Confidence Interval
OR: Odds Ratio

## References

[1] Soni SR (1974) Aneurysms of the posterior communicating artery and oculomotor paresis. J Neurol Neurosurg Psychiatry 37(4):475–484. 10.1136/jnnp.37.4.475 4838918 10.1136/jnnp.37.4.475 PMC494681

[2] Shree R, Mahesh KV, Balaini N, Goel A (2022) Oculomotor Cranial neuropathies: diagnosis and management. Ann Indian Acad Neurol 25(Suppl 2):S70–S82. 10.4103/aian.aian_167_22 36589037 10.4103/aian.aian_167_22 PMC9795710

[3] Kassis SZ, Jouanneau E, Tahon FB, Salkine F, Perrin G, Turjman F (2010) Recovery of third nerve palsy after endovascular treatment of posterior communicating artery aneurysms. World Neurosurg 73(1):11–16. 10.1016/j.surneu.2009.03.042 20452864 10.1016/j.surneu.2009.03.042

[4] Chalouhi N, Theofanis T, Jabbour P et al (2013) Endovascular treatment of posterior communicating artery aneurysms with Oculomotor nerve Palsy: clinical outcomes and predictors of nerve recovery. Am J Neuroradiol 34(4):828–832. 10.3174/ajnr.A3294 23042929 10.3174/ajnr.A3294 PMC7964498

[5] Gaberel T, Borha A, di Palma C, Emery E (2016) Clipping Versus coiling in the management of posterior communicating artery aneurysms with third nerve palsy: a systematic review and Meta-analysis. World Neurosurg 87:498–506e4. 10.1016/j.wneu.2015.09.026 26409080 10.1016/j.wneu.2015.09.026

[6] Date I, Asari S, Ohmoto T (1998) Cerebral aneurysms causing visual symptoms: their features and surgical outcome. Clin Neurol Neurosurg 100(4):259–267. 10.1016/S0303-8467(98)00047-X 9879851 10.1016/s0303-8467(98)00047-x

[7] Brigui M, Chauvet D, Clarençon F et al (2014) Recovery from oculomotor nerve palsy due to posterior communicating artery aneurysms: results after clipping versus coiling in a single-center series. Acta Neurochir (Wien) 156(5):879–884. 10.1007/s00701-014-2050-8 24610452 10.1007/s00701-014-2050-8

[8] Güresir E, Schuss P, Setzer M, Platz J, Seifert V, Vatter H (2011) Posterior communicating artery aneurysm–related Oculomotor nerve Palsy: influence of Surgical and Endovascular Treatment on Recovery: single-Center Series and systematic review. Neurosurgery 68(6):1527. 10.1227/NEU.0b013e31820edd82 21311376 10.1227/NEU.0b013e31820edd82

[9] Ko JH, Kim YJ (2011) Oculomotor nerve Palsy caused by posterior communicating artery aneurysm: evaluation of symptoms after Endovascular Treatment. Interv Neuroradiol 17(4):415–419 22192543 10.1177/159101991101700403 PMC3296499

[10] Chien A, Sayre J, Viñuela F (2013) Quantitative comparison of the dynamic flow waveform changes in 12 ruptured and 29 unruptured ICA–ophthalmic artery aneurysms. Neuroradiology 55(3):313–320. 10.1007/s00234-012-1108-7 23443738 10.1007/s00234-012-1108-7 PMC3582813

[11] Tan H, Huang G, Zhang T, Liu J, Li Z, Wang Z (2015) A retrospective comparison of the influence of Surgical clipping and endovascular embolization on recovery of Oculomotor nerve palsy in patients with posterior communicating artery aneurysms. Neurosurgery 76(6):687. 10.1227/NEU.0000000000000703 25786201 10.1227/NEU.0000000000000703

[12] Güresir E, Schuss P, Seifert V, Vatter H (2012) Oculomotor nerve palsy by posterior communicating artery aneurysms: influence of surgical strategy on recovery. J Neurosurg 117(5):904–910. 10.3171/2012.8.JNS111239 22937927 10.3171/2012.8.JNS111239

[13] Nam KH, Choi CH, Lee JI, Ko JG, Lee TH, Lee SW (2010) Unruptured intracranial aneurysms with Oculomotor nerve Palsy: clinical outcome between Surgical Clipping and Coil Embolization. J Korean Neurosurg Soc 48(2):109–114. 10.3340/jkns.2010.48.2.109 20856657 10.3340/jkns.2010.48.2.109 PMC2941851

[14] Zhang SH, Pei W, Cai XS, Cheng G (2010) Endovascular Management and Recovery from Oculomotor Nerve Palsy Associated with aneurysms of the posterior communicating artery. World Neurosurg 74(2):316–319. 10.1016/j.wneu.2010.05.036 21492565 10.1016/j.wneu.2010.05.036

[15] Mansour N, Kamel MH, Kelleher M et al (2007) Resolution of cranial nerve paresis after endovascular management of cerebral aneurysms. Surg Neurol 68(5):500–504. 10.1016/j.surneu.2006.12.061 17597189 10.1016/j.surneu.2006.12.061

[16] Ahn JY, Han IB, Yoon PH et al (2006) Clipping vs coiling of posterior communicating artery aneurysms with third nerve palsy. Neurology 66(1):121–123. 10.1212/01.wnl.0000191398.76450.c4 16401861 10.1212/01.wnl.0000191398.76450.c4

[17] Jha V, Sinha V, Abhijit V, Jha N, Singh S (2020) Comparative analysis of the risk factors influencing recovery of function from oculomotor nerve palsy in unruptured and ruptured posterior communicating artery aneurysms. Turk Neurosurg Published Online. 10.5137/1019-5149.JTN.32677-20.1 10.5137/1019-5149.JTN.32677-20.1 34664686

[18] Liu J, Peng C, Zhu G et al (2020) Comparison of surgical clipping and endovascular coiling in the treatment of oculomotor nerve palsy caused by posterior communicating artery aneurysm. Med (Baltim) 99(47):e22969. 10.1097/MD.0000000000022969 10.1097/MD.0000000000022969 PMC7676548 33217799

[19] gianni. Comparison of the efficacy of surgical clipping and embolization for oculomotor nerve palsy due to a posterior communicating artery aneurysm. European Review. January 27 (2017) Accessed January 19, 2024. https://www.europeanreview.org/article/12096 28165559

[20] Santillan A, Zink WE, Knopman J, Riina HA, Gobin YP (2010) Early Endovascular Management of Oculomotor Nerve Palsy Associated with posterior communicating artery aneurysms. Interv Neuroradiol 16(1):17–21. 10.1177/159101991001600102 20377975 10.1177/159101991001600102 PMC3277961

[21] Leivo S, Hernesniemi J, Luukkonen M, Vapalahti M (1996) Early surgery improves the cure of aneurysm-induced oculomotor palsy. Surg Neurol 45(5):430–434. 10.1016/0090-3019(95)00432-7 8629242 10.1016/0090-3019(95)00432-7

[22] Khan S, Agrawal A, Hailey C et al (2013) Effect of surgical clipping versus endovascular coiling on recovery from oculomotor nerve palsy in patients with posterior communicating artery aneurysms: a retrospective comparative study and meta-analysis. Asian J Neurosurg 8(03):117–124. 10.4103/1793-5482.121671 24403953 10.4103/1793-5482.121671 PMC3877497

[23] Wang B, Liu S, Na SJ et al (2022) Effects of endovascular treatment and prognostic factors for recovery of oculomotor nerve palsy caused by posterior communicating artery aneurysms: a multi-center retrospective analysis. BMC Neurol 22(1):380. 10.1186/s12883-022-02911-y 36209054 10.1186/s12883-022-02911-y PMC9547414

[24] Hu K, Cai G, Fu L et al (2021) Effects of surgical clipping and endovascular embolization on the recovery of oculomotor nerve paralysis caused by posterior communicating artery aneurysm. Neurol Asia 26(3):471–478. 10.54029/2021vnw

[25] Shen X, Wang W, Qin H, Ren CF, Gao BL (2022) Efficacy and long-term results of endovascular embolization and surgical clipping for posterior communicating artery unruptured aneurysms complicated with oculomotor nerve palsy. Med (Baltim) 101(34):e30421. 10.1097/MD.0000000000030421 10.1097/MD.0000000000030421 PMC9410630 36042618

[26] Sheehan MJ, Dunne R, Thornton J, Brennan P, Looby S, O’Hare A (2015) Endovascular repair of posterior communicating artery aneurysms, associated with oculomotor nerve palsy: a review of nerve recovery. Interv Neuroradiol 21(3):312–316. 10.1177/1591019915583222 26015520 10.1177/1591019915583222 PMC4757275

[27] Okauchi M, Matsumura H, Fujimori T et al (2022) Endovascular treatment for posterior communicating artery aneurysms with Oculomotor nerve Palsy. J Neuroendovascular Ther 16(5):243–249. 10.5797/jnet.oa.2021-0078 10.5797/jnet.oa.2021-0078 PMC10370555 37502228

[28] Shimoda K, Kano T, Kurata G, Kanazawa Y, Furuichi M, Yoshino A (2020) Endovascular treatment of patients with Oculomotor nerve Palsy Induced by posterior communicating artery aneurysms. J Neuroendovascular Ther 14(9):366–372. 10.5797/jnet.oa.2020-0001 10.5797/jnet.oa.2020-0001 PMC10370910 37501669

[29] Signorelli F, Pop R, Ganau M et al (2020) Endovascular versus surgical treatment for improvement of oculomotor nerve palsy caused by unruptured posterior communicating artery aneurysms. J NeuroInterventional Surg 12(10):964–967. 10.1136/neurintsurg-2020-015802 10.1136/neurintsurg-2020-015802 32139390

[30] Stiebel-Kalish H, Maimon S, Amsalem J, Erlich R, Kalish Y, Rappaport ZH (2003) Evolution of Oculomotor nerve paresis after endovascular coiling of posterior communicating artery aneurysms: a neuro-ophthalmological perspective. Neurosurgery 53(6):1268. 10.1227/01.NEU.0000093495.70639.AE 14633293 10.1227/01.neu.0000093495.70639.ae

[31] Lv N, Yu Y, Xu J, Karmonik C, Liu J, Huang Q (2016) Hemodynamic and morphological characteristics of unruptured posterior communicating artery aneurysms with oculomotor nerve palsy. J Neurosurg 125(2):264–268. 10.3171/2015.6.JNS15267 26636379 10.3171/2015.6.JNS15267

[32] Chen PR, Amin-Hanjani S, Albuquerque FC, McDougall C, Zabramski JM, Spetzler RF (2006) Outcome of Oculomotor nerve palsy from posterior communicating artery aneurysms: comparison of clipping and coiling. Neurosurgery 58(6):1040. 10.1227/01.NEU.0000215853.95187.5E 16723882 10.1227/01.NEU.0000215853.95187.5E

[33] Chang SI, Tsaı MD, Weı CP (2014) Posterior communicating aneurysm with Oculomotor nerve Palsy: clinical Outcome after Aneurysm Clipping. Turk Neurosurg.;24(2) 10.5137/1019-5149.JTN.6446-12.1 24831356

[34] Zhong W, Zhang J, Shen J et al (2019) Posterior communicating aneurysm with oculomotor nerve palsy: predictors of nerve recovery. J Clin Neurosci 59:62–67. 10.1016/j.jocn.2018.11.006 30455133 10.1016/j.jocn.2018.11.006

[35] Hou Y, Chen R, Yang H et al (2022) Predictors of complete recovery of oculomotor nerve palsy induced by posterior communicating artery aneurysms in patients aged eighteen to sixty. J Clin Neurosci 99:212–216. 10.1016/j.jocn.2022.03.015 35290936 10.1016/j.jocn.2022.03.015

[36] Abdurahman E, Amod K, Royston D, Harrichandparsad R (2020) Recovery of oculomotor nerve palsy after endovascular management of posterior communicating artery aneurysms. SA J Radiol 24(1):1887. 10.4102/sajr.v24i1.1887 32934839 10.4102/sajr.v24i1.1887 PMC7479415

[37] Zu QQ, Liu XL, Wang B et al (2017) Recovery of oculomotor nerve palsy after endovascular treatment of ruptured posterior communicating artery aneurysm. Neuroradiology 59(11):1165–1170. 10.1007/s00234-017-1909-9 28879505 10.1007/s00234-017-1909-9

[38] Patel K, Guilfoyle MR, Bulters DO et al (2014) Recovery of oculomotor nerve palsy secondary to posterior communicating artery aneurysms. Br J Neurosurg 28(4):483–487. 10.3109/02688697.2013.857007 24205923 10.3109/02688697.2013.857007

[39] Tian Lqiang, Fu Q (2020) xi. Recovery of posterior communicating artery aneurysm induced oculomotor nerve palsy: a comparison between surgical clipping and endovascular embolization. *BMC Neurol*.;20(1):351. 10.1186/s12883-020-01847-5 10.1186/s12883-020-01847-5 PMC7501645 32948136

[40] Gu DQ, Luo B, Zhang X, Long XA, Duan CZ (2012) Recovery of posterior communicating artery aneurysm-induced oculomotor nerve paresis after endovascular treatment. Clin Neurol Neurosurg 114(9):1238–1242. 10.1016/j.clineuro.2012.03.016 22464656 10.1016/j.clineuro.2012.03.016

[41] Hanse MCJ, Gerrits MCF, van Rooij WJ, Houben MPWA, Nijssen PCG, Sluzewski M (2008) Recovery of posterior communicating artery Aneurysm-Induced Oculomotor Palsy after coiling. Am J Neuroradiol 29(5):988–990. 10.3174/ajnr.A1019 18272550 10.3174/ajnr.A1019 PMC8128573

[42] Javalkar V, Cardenas R, Nanda A (2010) Recovery of third nerve Palsy following Surgical clipping of posterior communicating artery aneurysms. World Neurosurg 73(4):353–356. 10.1016/j.wneu.2010.01.002 20849792 10.1016/j.wneu.2010.01.002

[43] McCracken DJ, Lovasik BP, McCracken CE et al (2015) Resolution of Oculomotor nerve Palsy secondary to posterior communicating artery aneurysms: comparison of clipping and coiling. Neurosurgery 77(6):931. 10.1227/NEU.0000000000000965 26287555 10.1227/NEU.0000000000000965

[44] da Costa MDS, Lima JVF, Zanini MA et al (2023) Risks for Oculomotor nerve Palsy and Time to Recovery after Surgical clipping of posterior communicating artery aneurysms: a Multicenter Retrospective Cohort Study. Neurosurgery 92(6):1192. 10.1227/neu.0000000000002349 36752634 10.1227/neu.0000000000002349

[45] Park J, Kang DH, Chun BY (2011) Superciliary keyhole surgery for unruptured posterior communicating artery aneurysms with oculomotor nerve palsy: maximizing symptomatic resolution and minimizing surgical invasiveness: clinical article. J Neurosurg 115(4):700–706. 10.3171/2011.5.JNS102087 21699478 10.3171/2011.5.JNS102087

[46] Yang X, Zhou C, Liang L (2021) Surgical clipping and endovascular embolization for senile patients with posterior communicating artery aneurysms complicated with oculomotor nerve palsy. Am J Transl Res 13(5):5679–5684 34150176 PMC8205669

[47] Hall S, Sadek AR, Dando A et al (2017) The resolution of Oculomotor nerve Palsy caused by unruptured posterior communicating artery aneurysms: a Cohort Study and Narrative Review. World Neurosurg 107:581–587. 10.1016/j.wneu.2017.07.123 28765019 10.1016/j.wneu.2017.07.123

[48] Anan M, Nagai Y, Fudaba H et al (2014) Third nerve palsy caused by compression of the posterior communicating artery aneurysm does not depend on the size of the aneurysm, but on the distance between the ICA and the anterior–posterior clinoid process. Clin Neurol Neurosurg 123:169–173. 10.1016/j.clineuro.2014.05.006 24968189 10.1016/j.clineuro.2014.05.006

[49] Kameda-Smith M, Pai A, Jung Y et al (2022) Third nerve Palsy due to Intracranial aneurysms and Recovery after Endovascular Coiling. Can J Neurol Sci 49(4):560–568. 10.1017/cjn.2021.145 34167603 10.1017/cjn.2021.145

[50] Liberati A, Altman DG, Tetzlaff J et al (2009) The PRISMA statement for reporting systematic reviews and meta-analyses of studies that evaluate health care interventions: explanation and elaboration. J Clin Epidemiol 62(10):e1–e34. 10.1016/j.jclinepi.2009.06.006 19631507 10.1016/j.jclinepi.2009.06.006

[51] Covidence https://app.covidence.org/

[52] Luchini C, Stubbs B, Solmi M, Veronese N (2017) Assessing the quality of studies in meta-analyses: advantages and limitations of the Newcastle Ottawa Scale. World J Meta-Anal 5(4):80–84. 10.13105/wjma.v5.i4.80

[53] Cochrane Handbook for Systematic Reviews of Interventions. Accessed January 18 (2024) https://training.cochrane.org/handbook

[54] Higgins JPT, Thompson SG, Deeks JJ, Altman DG (2003) Measuring inconsistency in meta-analyses. BMJ 327(7414):557–560. 10.1136/bmj.327.7414.557 12958120 10.1136/bmj.327.7414.557 PMC192859

[55] Tufanaru C, Munn Z, Stephenson M, Aromataris E (2015) Fixed or random effects meta-analysis? Common methodological issues in systematic reviews of effectiveness. JBI Evid Implement 13(3):196. 10.1097/XEB.0000000000000065 10.1097/XEB.0000000000000065 26355603

[56] Pineda-Castillo SA, Jones ER, Laurence KA et al (2024) Systematic Review and Meta‐analysis of endovascular therapy effectiveness for Unruptured Saccular Intracranial aneurysms. Stroke Vasc Interv Neurol 4(2):e001118. 10.1161/SVIN.123.001118 38846323 10.1161/SVIN.123.001118 PMC11152505

[57] Kim MJ, Chung J, Park KY et al (2021) Recurrence and risk factors of posterior communicating artery aneurysms after endovascular treatment. Acta Neurochir (Wien) 163(8):2319–2326. 10.1007/s00701-021-04881-5 34143318 10.1007/s00701-021-04881-5

[58] Teasdale E, Statham P, Straiton J, Macpherson P (1990) Non-invasive radiological investigation for oculomotor palsy. J Neurol Neurosurg Psychiatry 53(7):549–553. 10.1136/jnnp.53.7.549 2391516 10.1136/jnnp.53.7.549 PMC488127

[59] Shimauchi-Ohtaki H, Tosaka M, Ohtani T et al (2018) Systemic metabolism and energy consumption after microsurgical clipping and endovascular coiling for aneurysmal subarachnoid hemorrhage. Acta Neurochir (Wien) 160(2):261–268. 10.1007/s00701-017-3400-0 29177598 10.1007/s00701-017-3400-0

