# Supplementary material for: Oculomotor nerve palsy recovery following microsurgery vs. endovascular treatment of posterior communicating artery aneurysms: a comparative meta-analysis of short- and long-term outcomes
Source: Neurosurg Rev. 2024 Dec 18;47(1):904. doi: 10.1007/s10143-024-03149-7 (PMC11655586; doi:10.1007/s10143-024-03149-7)
Supplement: Supplementary file 1 — Supplementary Material 1 [file 10143_2024_3149_MOESM1_ESM.docx]

**Supplemental material 1, table 1: Baseline characteristics of included 21 non-comparative studies.** NOS: Newcastle-Ottawa Quality Assessment Scale, SD: Standard Deviation, ONP: Oculomotor Nerve Palsy, EVT: Endovascular Therapy.

| Authors, Year | Journal | Quality assessment (modified NOS^*^): (score, quality) | Treatment Modality | Sample Size, n | Females, n (%) | Age, years (mean±SD) | ONP at presentation, n(%) | | Rupture Status, n(%) | | Aneurysm size, mm (mean±SD) | Follow up intervals (months) | last follow up Outcomes (favorable recovery) n(%) |
| --- | --- | --- | --- | --- | --- | --- | --- | --- | --- | --- | --- | --- | --- |
|  |  |  |  |  |  |  | Partial | Total | Unruptured | Ruptured |  |  |  |
| Kameda-Smith 2022^49^ | Canadian Journal of Neurological Sciences | 6, high | EVT | 37 | 34(92) | NA | 30(81) | 7(19) | 20(54) | 17(46) | 9.48(±7.2) | NA | 20(54) |
| Okauchi 2022^27^ | J Neuroendovasc Ther | 7, high | EVT | 23 | 21(91) | 72.7(±15) | 9(39) | 14(61) | 14(61) | 9(39) | 9.1(±3.5) | 1, 3, 6, 12, 18, >24 | 20(87) |
| Wang 2022^23^ | BMC Neurol | 8, high | EVT | 211 | 176(83) | 60.8(±11.2) | 126(60) | 85(40) | 97(46) | 114(54) | 6.5(±2.7) | 6 | 126(60) |
| Abdurahman 2021^36^ | South African Journal of Radiology | 7, high | EVT | 34 | 22(65) | 49.8(±10.1) | 2(6) | 32(94) | 7(21) | 27(79) | 6.7(±3.1) | 12 | 30(88) |
| Shimoda 2020^28^ | J Neuroendovasc Ther | 7, high | EVT | 8 | 7(88) | 73(±13) | 2(25) | 6(75) | 8(100) | 0 | 7.9(±1.25) | NA | 4(50) |
| Zu 2017^37^ | Neuroradiology | 7, high | EVT | 34 | 29(85) | NA | 22(65) | 12(35) | 0 | 34(100) | NA | 6 | 29(85) |
| Sheehan 2015^26^ | Interv Neuroradiol | 6, high | EVT | 20 | 12(60) | NA | 11(55) | 9(45) | 13(65) | 7(35) | 6.89(±1.9) | 1, 3, 6, 12, 18, >24 | 9(45) |
| Chalouhi 2013^4^ | AJNR Am J Neuroradiol | 8, high | EVT | 37 | 29(78) | 54.8(±12) | 18(49) | 19(51) | NA | NA | 7.6(±3.2) | NA | 33(89) |
| Gu 2012^40^ | Clin Neurol Neurosurg | 7, high | EVT | 36 | 20(56) | 54.3(±9) | 22(61) | 14(39) | 15(42) | 21(58) | 9.3(3.9) | NA | 17(47) |
| Ko 2011^9^ | Interv Neuroradiol | 7, high | EVT | 10 | 9(90) | 62.7(±9.3) | 6(60) | 4(40) | 1(10) | 9(90) | 9.7(2.9) | 1, 3 | 8(80) |
| Kassis 2010^3^ | World Neurosurg | 8, high | EVT | 20 | 18(90) | 51.4(±13) | 12(60) | 8(40) | 9(45) | 11(55) | 7(1.2) | 1, 3, 6, 12, 18 | 7(35) |
| Zhang 2010^14^ | World Neurosurg | 7, high | EVT | 13 | 13(100) | 49.6(±9.7) | 5(38) | 8(62) | 5(38) | 8(62) | 7.9(±2.8) | NA | 8(62) |
| Santillan 2010^20^ | Interv Neuroradiol | 5, moderate | EVT | 11 | 10(91) | 52.6 | 5(45) | 6(55) | 8(73) | 3(27) | 7.7(±2.0) | NA | 7(64) |
| Hanse 2008^41^ | AJNR Am J Neuroradiol | 6, high | EVT | 21 | 19(90) | 54.9 | 6(29) | 15(71) | 4(19) | 17(81) | 9(±1.0) | NA | 8(38) |
| Stiebel-Kalish 2003^30^ | Neurosurgery | 5, moderate | EVT | 11 | NA | NA | 0 | 11(100) | 1(9) | 10(91) | 10.5(±4.1) | 3, 12 | 4(36) |
| daCostaMDS 2023^44^ | Neurosurgery | 6, high | Microsurgery | 119 | 101(84.9) | 56(±1.5) | NA | NA | 31(26) | 88(74) | 7.5(±1) | NA | 100(84) |
| Chang 2014^33^ | Turk Neurosurg | 7, high | Microsurgery | 10 | 8(80) | 59.9(±10.6) | 8(80) | 2(20) | 7(70) | 3(30) | NA | NA | 7(70) |
| Park 2011^45^ | Neurosurgery | 7, high | Microsurgery | 13 | NA | 51.5(±11) | 6(46) | 7(54) | 13(100) | 0 | 8.4(±1.7) | 1, 3, 6 | 12(92) |
| Javalkar 2010^42^ | World Neurosurg | 5, moderate | Microsurgery | 26 | 21(81) | 53 | 8(31) | 18(69) | 5(19) | 21(81) | NA | NA | 20(77) |

^*^  **Modified NOS:** Comparability (point a) was not tested because of the design of the reported studies. Comparability (point b) was tested comparing subgroups of analysis: One point was attributed if the study reported the analysis of the subgrou

**Supplemental material 2, table 2:** **Baseline characteristics of included 19 comparative studies.** NOS: Newcastle-Ottawa Quality Assessment Scale, SD: Standard Deviation, ONP: Oculomotor Nerve Palsy, EVT: Endovascular Therapy.

| Authors, Year | Journal | Quality assessment (NOS): (score, quality) | Sample Size, n | | | Females, n (%) | | Age, years (mean±SD) | | ONP at presentation, n(%) | | Rupture Status, n(%) | | Aneurysm size, mm (mean±SD) | | Follow up (months) | last follow up Outcomes (favorable recovery) n(%) | |
| --- | --- | --- | --- | --- | --- | --- | --- | --- | --- | --- | --- | --- | --- | --- | --- | --- | --- | --- |
|  |  |  | Total | Microsurgery | EVT | Microsurgery | EVT | Microsurgery | EVT | Partial | Total | Unruptured | Ruptured | Microsurgery | Endovascular |  | Microsurgery | EVT |
| Hou 2022^35^ | J Clin Neurosci | 8, high | 88 | 60 | 28 | NA | NA | NA | NA | 43(49) | 45(51) | 63(72) | 25(28) | NA | NA | 12 | 46(77) | 16(57) |
| Shen 2022^25^ | Medicine (Baltimore) | 7, high | 80 | 40 | 40 | 18(45) | 17(43) | 53.45(±3.29) | 52.16 (± 3.54) | 29(36) | 51(64) | NA | NA | 8.1(± 1.4) | 7.8(± 1.2) | 1, 3, 6, 12, 18 | 39(98) | 38(95) |
| Yang 2021^46^ | Am J Transl Res | 8, high | 96 | 48 | 48 | 20(42) | 23(48) | NA | NA | NA | NA | NA | NA | NA | NA | 12 | 28(58) | 40(83) |
| Hu 2021^24^ | Neurology Asia | 7, high | 128 | 96 | 32 | 53(55) | 15(47) | 53.6(±11.7) | 52.8(±10.2) | 66(52) | 62(48) | 65(51) | 63(49) | 7.82(±1.70) | 7.71(± 1.63) | 12 | 94(98) | 32(100) |
| Jha 2021^17^ | Turk Neurosurg | 8, high | 25 | 14 | 11 | 7(50) | 7(64) | 49.7(±8.42) | 51.17(±10.46) | 13(52) | 12(48) | 13(52) | 12(48) | NA | NA | 12 | 13(93) | 10(91) |
| Tian 2020^39^ | BMC Neurol | 9, high | 70 | 31 | 39 | 17(55) | 18(46) | NA | NA | 15(21) | 55(79) | 62(89) | 8(11) | 7.6(± 2,9) | 7.9 (± 3,0) | 12 | 26(84) | 25(64) |
| Signorelli 2020^29^ | J Neurointerv Surg | 6, moderate | 55 | 24 | 31 | NA | NA | NA | NA | 28(51) | 27(49) | 55(100) | 0 | NA | NA | > 24 | 21(88) | 19(61) |
| Liu 2020^18^ | Medicine (Baltimore) | 8, high | 152 | 112 | 40 | 62(55) | 20(50) | 56.2(± 8.2) | 55.6(± 7.9) | 77(51) | 75(49) | 75(49) | 77(51) | 7.91(± 1.23) | 7.65(±1.58) | 12 | 106(95) | 26(65) |
| Zhong 2019^34^ | J Clin Neurosci | 7, high | 102 | 39 | 63 | 32(82) | 53(84) | 54.7(± 9.9) | 62.9(± 9.7) | 40(39) | 62(61) | 55(54) | 47(46) | 6.90(± 2.87) | 6.90(± 2.56) | > 24 | 39(100) | 61(97) |
| MakSKD 2018^57^ | Asian J Neurosurg | 7, high | 22 | 11 | 11 | 7(64) | 9(82) | 57.9 | 59.6 | 9(41) | 13(59) | 11(50) | 11(50) | NA | NA | 1, 3, 6, 12 | 11(100) | 9(82) |
| Hall 2017^47^ | World Neurosurg | 8, high | 15 | 5 | 10 | NA | NA | NA | NA | NA | NA | 15(100) | 0 | NA | NA | 6 | 3(60) | 1(10) |
| Gao 2017^19^ | Eur Rev Med Pharmacol Sci | 6, moderate | 52 | 23 | 29 | 16(69.56) | 28(96.5) | 53.9±11.5 | 54.1±10.0 | 7(13) | 45(87) | 18(34.62) | 34(65.38) | 7.1(±2.1) | 6.4(±1.8) | 12 | 23(100) | 25(86) |
| McCracken 2015^43^ | Neurosurgery | 7, high | 93 | 70 | 23 | 61(87.14) | 22(95.65) | NA | NA | 61(66) | 32(34) | 38(40.86) | 55(59.13) | 7.0(3.1) | 6.1(±2.1) | 1, 3 | 40(57.14) | 10(43) |
| Tan 2015^11^ | Neurosurgery | 7, high | 176 | 132 | 44 | 75(56.82) | 21(47.73) | 51.73±9.66 | 54.65±12.13 | 88(50) | 88(50) | 87(49.43) | 89(50.57) | 7.80(±1.73) | 7.69(±1.70) | 12 | 130(98.48) | 30(68) |
| Patel 2014^38^ | Br J Neurosurg | 7, high | 18 | 9 | 9 | 7(77.7 | 8(88.88) | 52.3±16.4 | 67.7±12.6 | 5(28) | 13(72) | 3(16.67) | 15(83.33) | 8.0(±1.1) | 7.5(±1.5) | > 24 | 6(66.66) | 5(56) |
| Brigui 2014^7^ | Acta Neurochir (Wien) | 9, high | 21 | 7 | 14 | 5(71.43) | 11(78.57) | 60.7±10.3 | 49.2±11.0 | 8(38) | 11(52) | 5(23.80) | 16(76.19) | 7.3(±3.8) | 8.3(±2.9) | > 24 | 6(85.71) | 8(57) |
| Khan 2013^22^ | Asian J Neurosurg | 8, high | 17 | 8 | 9 | 8(100) | 8(88.88) | NA | NA | 10(59) | 7(41) | 11(64.71) | 6(35.29) | 6.5(±1.2) | 7.4(±3.8) | 12 | 7(87.5) | 4(44) |
| Guresir 2011^8^ | Neurosurgery | 8, high | 11 | 4 | 7 | NA | NA | NA | NA | 4(36) | 7(64) | 6(54.54) | 5(45.45) | 9(±5) | 7(±1) | 12 | 4(100) | 3(43) |
| Nam 2010^13^ | KOREAN NEUROSURGICAL | 7, high | 19 | 9 | 10 | 7 (77.78) | 8 (80.00) | 57.4(±14.0) | 55.8(±12.4) | 5(26) | 14(74) | 19(100) | 0(0) | 6.1(±2.1) | 12.8(±6.4) | 6 | 8(89) | 7(70) |
| Chen 2006^32^ | Neurosurgery | 7, high | 13 | 7 | 6 | 6 (85.71) | 6 (100.00) | 53.9(±14.6) | 57.3(15.5) | 6(46) | 7(54) | 4(31) | 9(69) | 8.6(±3.6) | 6.0(±1.2) | 12, 18 | 6(86) | 2(33) |
| Ahn 2006^16^ | Neurology | 6, unclear | 17 | 7 | 10 | 7 (100.00) | 10 (100.00) | NA | NA | 4(24) | 13(76) | 17(100) | 0(0) | 9.5(±3.8) | 8.6(4.1) | 18 | 3(43) | 6(60) |

**Supplemental material 3, table 3: Subgroup Analysis of Favorable ONP Recovery Outcomes: Inclusive of Pooled Data from Both Comparative and Single-Arm Studies on PComA Aneurysms Associated with ONP.** ONP: Oculomotor Nerve Palsy

| **Subgroups** | **Microsurgery** | | | **Endovascular** | | | **Subgroup differences (favorable ONP recovery outcomes according to treatment)** | | **Subgroup differences (favorable recovery ONP outcomes according baseline groups)** | | |
| --- | --- | --- | --- | --- | --- | --- | --- | --- | --- | --- | --- |
|  | **Proportion. Raw, overall (%)** | **95%-CI** | **Heterogeneity: I^2^(%), (P-Value).** | **Proportion. Overall (%)** | **95%-CI** | **Heterogeneity: I^2^(%), (P-Value)** | **Heterogeneity: I^2^(%), (P-Value)** | **Test for subgroup differences. P-Value** | **Proportion. Raw Overall (%)** | **Heterogeneity: I^2^(%), (P-Value)** | **Test for subgroup differences. P-Value** |
| Patients ≥60-year-old | 54/82, (65.90) | [0.53-0.74] | 0, 0.74 | 125/176, (71.0) | [0.51-0.76] | 52, 0.01 | 36, 0.05 | 0.92 | 179/258, (69.38) | 33, 0.02 | 0.76 |
| Patients <60-year-old | 81/99, (81.82) | [0.69-0.86] | 0, 0.62 | 90/141, (63.83) | [0.51-0.73] | 25, 0.19 | 32, 0.08 | 0.03 | 171/240, (71.25) |  |  |
| Partial ONP | 81/108, (75.00) | [0.65-0.75] | 0, 1.00 | 211/294, (71.77) | [0.60-0.75] | 10, 0.32 | 0, 0.81 | 0.27 | 292/402, (72.64) | 47, <0.01 | **<0.01** |
| Total ONP | 93/134, (69.40) | [0.57-0.77] | 10, 0.34 | 157/324, (48.46) | [0.37-0.61] | 63, <0.01 | 58, <0.01 | 0.02 | 250/458, (54.59) |  |  |
| Unruptured aneurysm | 83/112, (74.11) | [0.57-0.87] | 46, 0.06 | 133/252, (52.78) | [0.43-0.52] | 34, 0.07 | 46, <0.01 | 0.03 | 216/364, (59.34) | 44, <0.01 | 0.39 |
| Ruptured aneurysm | 51/69, (73.91) | [0.59-0.81] | 0, 0.59 | 209/315, (66.35) | [0.52-0.71] | 45, 0.02 | 34, 0.05 | 0.23 | 260/384, (67.71) |  |  |
| Small aneurysm ≤ 7 mm | 45/57, (78.95) | [0.58-0.84] | 0, 0.55 | 114/176, (64.77) | [0.50-0.73] | 42, 0.03 | 32, 0.06 | 0.24 | 159/233, (68.23) | 21, 0.10 | 0.88 |
| Large aneurysm > 7 mm | 36/42, (85.71) | [0.67-0.90] | 0, 0.91 | 83/136, (61.03) | [0.48-0.69] | 9, 0.34 | 8, 0.34 | 0.01 | 119/178, (66.85) |  |  |
| Early treatment ≤ 7 days | 58/73, (79.45) | [0.64-0.85] | 0, 0.81 | 227/327, (69.40) | [0.58-0.76] | 33, 0.09 | 19, 0.20 | 0.24 | 319/435, (73.33) | 0, 0.70 | **<0.01** |
| Late treatment > 7 days | 62/100, (62.00) | [0.55-0.87] | 70, <0.01 | 81/168, (48.21) | [0.22-0.54] | 85, < 0.01 | 39, <0.01 | < 0.01 | 209/335, (62.39) |  |  |
